# Role of Defective Interfering Particles in Complement-Mediated Lysis of Parainfluenza Virus-Infected Cells

**DOI:** 10.3390/v17040488

**Published:** 2025-03-28

**Authors:** Jenna R. Aquino, Candace R. Fox, Griffith D. Parks

**Affiliations:** Burnett School of Biomedical Sciences, College of Medicine, University of Central Florida, Orlando, FL 32827, USA; jenna.aquino@ucf.edu (J.R.A.); candace.fox@ucf.edu (C.R.F.)

**Keywords:** defective interfering particles, defective viral genomes, parainfluenza virus type 5, complement, interferon-beta

## Abstract

RNA viruses pose a significant global public health burden due to their high mutation rates, zoonotic potential, and ability to evade immune responses. A common aspect of their replication is the generation of defective interfering particles (DIPs), which contain truncated defective viral genomes (DVGs) that depend on full-length standard (STD) virus for replication. DVGs have gained recognition as they are increasingly detected in clinical samples from natural infections. While their role in modulating type I interferon (IFN-I) responses is well established, their impact on the complement (C′) system is not understood. In this study, we examined how DVGs influence C′-mediated lysis during parainfluenza virus 5 (PIV5) infection using real-time in vitro cell viability assays. Our results demonstrated that C′ effectively killed human lung epithelial cells infected with STD PIV5, whereas co-infection with DIP-enriched stocks significantly suppressed C′-mediated killing through mechanisms that were dependent on DVG replication but independent of IFN-I production. The titration of DI units in co-infection with STD PIV5 showed a strong linear relationship between DIP-mediated decreases in surface viral glycoprotein expression and the inhibition of C′-mediated lysis. Our findings reveal a previously unrecognized function of DVGs in modulating C′ pathways, shedding light on their potential role in viral persistence and immune evasion.

## 1. Introduction

RNA viruses pose important public health challenges both in the United States and globally due to their rapid mutation rates, zoonotic potential, frequency of emergence and re-emergence, and capacity to evade host immune defenses [1,2,3,4]. These properties create formidable challenges for the effective management and treatment of viral infections. Diseases caused by RNA viruses, such as COVID-19, influenza, and AIDS, impact millions of lives through acute and chronic viral infections that drive significant mortality, lasting health complications, and the burden of high healthcare costs [5,6]. 

Historically, RNA viruses are known to generate defective interfering particles (DIPs) during cell culture propagation—truncated defective viral genomes (DVGs) that co-exist in the virus population along full-length standard (STD) virus [7,8]. DVGs are considered “defective” because they typically lack large parts of the viral genome but preserve essential regulatory elements, such as promoter and assembly sequences, needed for replication by the viral RNA-dependent-RNA polymerase (RdRp) and for packaging into progeny virions. Negative-strand RNA viruses generate two general types of DVGs: deletion-type, which results from the loss of an internal portion of the viral genome, and copyback-type, which arises when the viral polymerase falls off the antigenomic RNA template and rebinds to the nascent complementary genomic RNA segment emerging from the polymerase. This creates a panhandle loop structure with complementary ends [7]. Since these defective genomes lack vital viral coding regions, they are “interfering” because they rely on the helper STD virus to supply trans-acting proteins for their growth, creating cycles of viral competition for factors [8].

DIPs have been viewed in the past as simple byproducts of viral replication in cell culture, raising the question of their relevance to natural human infections. However, there is increasing evidence of the presence of DVGs in various natural infections [9,10,11,12,13,14,15,16,17,18,19,20,21,22]. Notably, clinical samples from patients who experienced neurological complications related to persistent measles virus infections, such as subacute sclerosing panencephalitis (SSPE), show significantly elevated levels of DVGs [14,18]. Additionally, DIPs have been shown to frequently emerge during the production of vaccines that are derived from live-attenuated viruses, including measles virus, influenza virus, and poliovirus. Research on their potential impact on vaccine efficacy remains limited [23,24,25,26].

DVGs can be strong inducers of type I interferons (IFN-I) through their production of potent pathogen-associated molecular patterns (PAMPs), such as double-stranded RNA (dsRNA), that are recognized by host innate immune sensors like retinoic acid-inducible gene I (RIG-I) and melanoma differentiation-associated protein 5 (MDA5) [27,28,29,30,31,32,33,34]. The binding of these receptors to DVG replication products can trigger downstream activation of mitochondrial antiviral signaling protein (MAVS), leading to the increased expression of IFN-I and subsequent activation of other IFN-stimulated genes (ISGs). With a landscape of hundreds of ISGs potentially activated by DVGs, it is clear that DVG infections can profoundly change the level of virus gene expression and growth, the intracellular environment, the surface of infected cells, and the innate immune responses [35]. In addition to IFN-I induction, DVG infections have recently been shown to drive pro-survival signals through TNF-alpha production [36], a mechanism proposed to contribute to the persistent infection of DVG infected cells. 

Complement (C′) is a powerful innate antiviral system [37,38] which, upon activation, leads to the recognition of microbes, direct neutralization of viruses, lysis of infected cells, and recruitment and activation of lymphocytes [39,40]. C′ factors sense viruses or virus-infected cells through three distinct pathways: classical, lectin, and alternative. These pathways converge on a central component C3 that triggers the formation of factors C5–C9 into the membrane attack complex (MAC), resulting in cell lysis of the infected cell or direct opsonization of viral particles. Importantly, C′ can be activated by many mechanisms, including changes in cell surfaces, exposure to damaged cells, expression of PAMPs, and detection of viral antigens on cell surfaces. The role of DVGs in either promoting or dampening C′ reactions to virus infection is largely unknown.

Given the potential for DVGs to change STD virus gene expression, induce IFN-I synthesis, change cell surfaces, and promote cell survival pathways, we tested the hypothesis that DVGs would alter the ability of C′ to lyse virus infected cells. To test this, we utilized a prototypic negative-strand RNA virus, parainfluenza virus 5 (PIV5), which frequently generates DVGs due to its RdRp error-prone replication [41,42]. Using DIP-enriched stocks of PIV5 generated in cell culture, our results show that STD PIV5 infection is a potent inducer of C′-mediated cell killing but, importantly, this cell killing is dramatically inhibited by co-infection with DIPs. The mechanism of quenching C′-mediated cell killing is dependent on DVG replication but independent of IFN-I production. These findings reveal the complexity of roles for DVGs in C′-mediated lysis, posing challenges for therapies aimed at clearing prolonged viral infections or enhancing innate antiviral immune responses.

## 2. Materials and Methods

### 2.1. Cells Lines, Viruses, and Infections

Cultures of CV1, Vero, A549, H1299, and Hep2 (ATCC) cells were grown in Dulbecco-modified Eagle medium (DMEM, Gibco, Thermo Fisher Scientific, Waltham, MA, USA) supplemented with 10% heat-inactivated fetal calf serum (HI-FBS, Gibco, Thermo Fisher Scientific) as previously described [43]. H1975 cultures (NCI-H1975 cells, catalog #: CRL-5908; ATCC) were grown in Roswell Park Memorial Institute medium (RPMI, Gibco, Thermo Fisher Scientific, Waltham, MA, USA) supplemented with 10% HI-FBS. Commercially available A549-MAVS-KO cells (A549-Dual™ KO-MAVS cells, catalog #:a549d-komavs; InvivoGen, San Diego, CA, USA) were grown in DMEM containing 10% HI-FBS, 100 U/mL penicillin, 100 µg/mL streptomycin, and 100 µg/mL Normocin. Transduction of cells using NucLight Red lentivirus (Sartorius, Göttingen, Germany), followed by 1 µg/mL puromycin selection, was used to generate A549, H1299, Hep2, and H1975 cells expressing a nuclear red fluorescence protein (A549-NLR, H1299-NLR, Hep2-NLR, H1975-NLR cells).

Wild-type PIV5 encoding a hemagglutinin (HA)- tagged version of thymidine kinase (TKHA) and the Leader (Le) mutant PIV5 encoding Green Fluorescent Protein (GFP) inserted between the HN and L were grown in Vero cells as previously described and titered on CV-1 cells [41,44,45]. These two versions of PIV5 are referred to here as Standard Virus (STD). Cells were infected at a multiplicity of infection (MOI) of 10 unless otherwise indicated and cultured in DMEM supplemented with 2% HI-FBS.

### 2.2. Generation of Defective Interfering Particle-Enriched Viral Stocks

To generate stocks enriched in Defective Interfering Particles (DIPs), undiluted serial passage of Le PIV5 was carried out in Vero cells as previously described [46]. Briefly, naïve cells were seeded to reach 80–90% confluency and infected with PIV5. Every two days, the media was collected and either frozen for further analysis or used immediately for the undiluted infection of naïve Vero cells. This serial passaging method was repeated across multiple cycles, and aliquots from each passage were stored for further analysis. Levels of standard virus contained in these serially passaged viral samples were identified by Hemagglutination assay, as previously described, using chicken red blood cells (Lampire, catalog #7241409, Pipersville, PA, USA) [47]. When the HA titers from each passage were plotted, stocks exhibiting the von Magnus effect, characterized by oscillating peaks in HA activity across passages, were selected. Stocks that showed consistently low HA titers were indicative of higher DIP enrichment, as these reflect a decreased proportion of STD PIV5 particles. These samples were selected and utilized as DIP-enriched viral stocks in subsequent experiments.

### 2.3. Cytotoxicity and Cell Killing Assays

Real-time cell viability assays were performed using an IncuCyte instrument (Sartorious, Göttingen, Germany) as previously described [39,40]. Briefly, target NLR cells were plated in triplicate in 96-well plates (Corning, New York, NY, USA) at 7000 cells/well and incubated overnight. Cells were mock-infected, or infected with STD PIV5, DIP-stock, or STD PIV5 plus DIP co-infected for 18 hprior to the addition of normal human serum (NHS; Innovative Research, Novi, MI, USA) as a source of C′ factors. Heat-inactivated (HI) sera were prepared by heating at 56 °C for 30 min to inactivate C′. Plates of cells were incubated at 37 °C within the IncuCyte system and imaged every 2 h using a 10× objective with red, green, and phase channels. The red object count (ROC) corresponding to cell nuclei was calculated for each well. The values for the ROC at each timepoint were expressed as a percentage of the value at time zero (ROCt0). The percentage cytotoxicity was calculated by normalizing the ROC of the wells containing NHS plus target cells to the ROC of the wells containing target cells treated with HI NHS.

### 2.4. Flow Cytometry

Cells cultured in 96-well plates were mock-infected or infected with STD PIV5-TKHA or STD PIV5-TKHA + DIP at a range of DIP concentrations. At 18 h post infection (hpi), media and trypsinized adherent cells were centrifuged, and either fixed and permeabilized for intracellular staining using Invitrogen eBioscience (Thermo Fisher Scientific) reagents according to manufacturer’s recommendations or processed directly for cell surface staining. Samples were intracellularly stained using anti-HA Tag monoclonal antibody (clone 2-2.2.14) (1:500 dilution, catalog #: 26183; Thermo Fisher Scientific, Waltham, MA, USA) or surface stained using a mouse anti-PIV5 polyclonal antibody (1:100 dilution, [48]). This was followed by secondary staining by anti-mouse AlexaFluor 405 (1:1000, catalog #: A31553; Thermo Fisher Scientific, Waltham, MA, USA). FITC channel was utilized on the flow cytometer to monitor infection for viruses that contain a GFP transgene and evaluate the interference capability of DIP-enriched stocks. All cell experiments were quantified by flow cytometry using the CytoFLEX (Beckman Coulter, Brea, CA, USA) and 10,000 independent events were analyzed using CytExpert software (Beckman Coulter, version 2.4).

### 2.5. Western Blotting

Cell lysates were resolved on 12% sodium dodecyl sulfate-polyacrylamide gel electrophoresis (SDS-PAGE) gels and transferred to nitrocellulose membranes (Bio-Rad, Hercules, CA, USA). After normalizing the samples by Western blotting for β-actin (1:10,000 dilution, catalog #: A5316; Sigma-Aldrich, St. Louis, MO, USA), samples were probed with antibodies for the HA Tag Monoclonal Antibody (clone 2-2.2.14) and PIV5 P protein (1:500 dilution, [49]). Blots were visualized using secondary horseradish peroxidase (HRP)-conjugated antibodies (Sigma-Aldrich) and chemiluminescence (Thermo Fisher Scientific).

### 2.6. Quantitative-PCR

Six-well plates of cells were treated as indicated in the figure legends, and RNA extraction was performed using TRIzol (Invitrogen, Carlsbad, CA, USA) as described previously [50]. TaqMan^®^ Reverse Transcription Reagents (Applied Biosystems, Foster City, CA, USA) were used to obtain cDNA from 1 µg of total RNA (as per the manufacturer’s instructions). Bio-Rad CFX Connect Real-Time and SYBR^®^ FAST Green Master Mix (Applied Biosystems) were used to perform quantitative real-time PCR. The primer sequences utilized are shown in Table 1, below.

### 2.7. ELISA

Dishes of cells were treated as detailed in the figure legends and media were evaluated using a Human IFN-β ELISA kit (catalog #: 41410-2; PBL Assay Science, Piscataway, NJ, USA) as described by the manufacturer. ELISA results were normalized to 10^6^ cells.

### 2.8. Statistics

All statistical analyses were performed using Prism GraphPad. Groupwise comparisons were assessed using Student’s *t*-test (2 groups) or analysis of variance (ANOVA; more than 2 groups). In all figures, * indicates a *p* value < 0.05, ** indicates a *p* value < 0.01, *** indicates a *p* value < 0.001, and **** indicates a *p* value < 0.0001.

## 3. Results

### 3.1. Generation of DIP-Enriched PIV5 Stocks and RNA Sequence Analysis of DVG Structures

**Figure 1:**
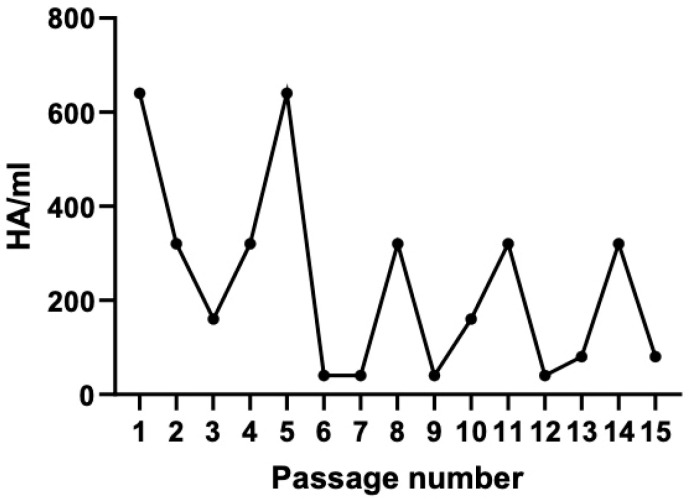
Generation of DIP-enriched PIV5 Stocks. An STD PIV5 infection of Vero cells was used to initiate rounds of serial undiluted passage of virus through Vero cells. At each cell pass, media was harvested and titered (HA/mL) by hemagglutination assay. Viral stocks with titers below 200 HA/mL were considered DIP-enriched PIV5 stocks and utilized for subsequent experiments.

To generate PIV5 stocks enriched in DIPs, undiluted serial passage of PIV5 was carried out in Vero cells as previously described [46]. In this study, the PIV5 Leader mutant expressing GFP was utilized as the parental “STD virus” for DIP-stock generation, since we have shown previously that this virus is a strong activator of the C′ system [39,40,41]. At each virus pass in culture, the media was harvested and assayed by hemagglutination (HA) assay for levels of STD virus. As shown in Figure 1, PIV5 viral stocks generated by undiluted serial passage showed the expected previously described oscillating peaks of high and low HA units, consistent with the von Magnus effect commonly seen with DIP generation in tissue culture [51]. Stocks with low viral HA titer were selected as those enriched in DIPs for subsequent experiments (e.g., passes 6, 7, 9, 12).

### 3.2. PIV5 DIPs Suppress STD Virus Gene Expression and Reduce Expression of PIV5 Surface Glycoproteins During Co-Infection

**Figure 2:**
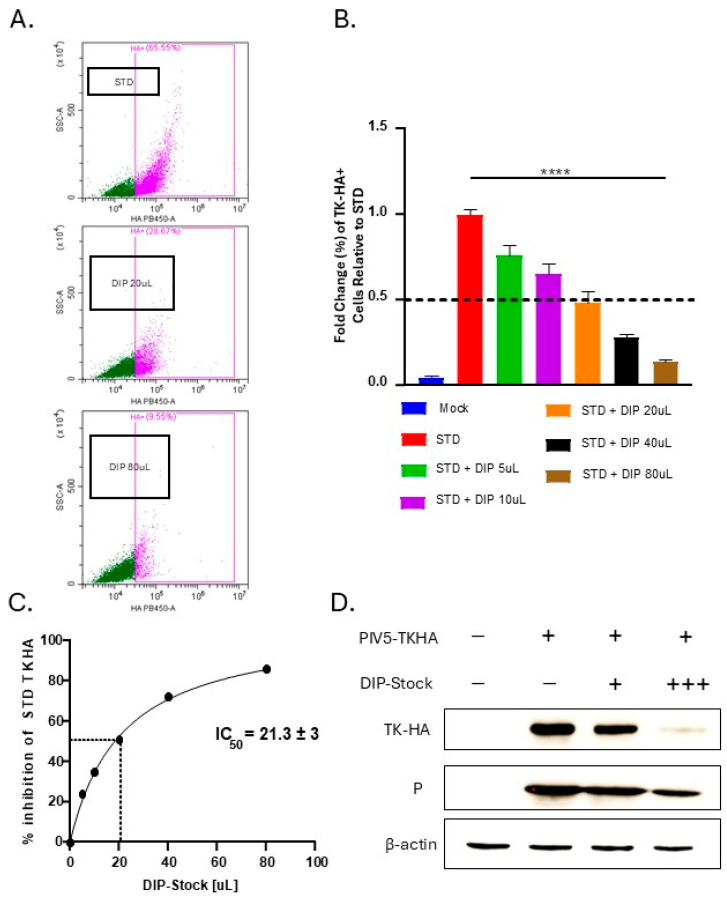
Establishing a functional unit for quantifying DIP-induced inhibition of STD PIV5 viral protein levels. (**A**–**D**) A549 cells were mock-infected, infected with STD PIV5-TKHA at an MOI of 10, or co-infected with STD PIV5-TKHA and increasing doses of DIP-enriched stock (1, 5, 10, 20, 40, 80 μL). At 18 hpi, levels of TKHA were determined by cell staining with an anti-HA antibody and flow cytometry. (**A**) Representative TKHA staining scatterplot images comparing STD PIV5-TKHA-infected cells to co-infections of STD PIV5-TKHA with DIP-enriched stock at 20 and 80 μL doses. (**B**) Fold change (%) in TKHA-positive cells relative to STD PIV5-TKHA. (**C**) Relationship between amount of DIP-enriched stock and inhibition of STD PIV5-TKHA expression. The IC50 was determined to be 21.3 μL in a 60,000-cell population, defined as 1 Defective Interfering Unit (DI unit). (**D**) A549 cells were mock-infected, infected with STD PIV5-TKHA infected at an MOI of 10, or co-infected with STD PIV5-TKHA and DIP-enriched virus with 0.25 (low dose) or 1 (high dose) DI unit. At 18 hpi, cell lysates were analyzed by Western blot for levels of TKHA and the PIV5 P protein.

Titration experiments were carried out to define a functional DIP unit as the amount of DIP stock required to reduce viral protein levels from STD full-length virus by 50 percent. A549 lung epithelial cells were mock-infected, infected with STD PIV5 expressing the marker gene TKHA at an MOI of 10, or co-infected with STD PIV5-TKHA and increasing doses of DIP-enriched stock. Levels of TKHA expression were quantified at 18 hpi by flow cytometry with an anti-HA antibody. As shown in representative Figure 2A, infection with STD PIV5-TKHA resulted in a strong expression of TKHA in 65% of cells, and this was decreased by co-infection with 20 μL and 80 μL of DIP-enriched stock. When examined for all doses of DIP in co-infections (Figure 2B), a dose-dependent decrease in STD PIV5-TKHA protein expression was observed across 5 μL to 80 μL of DIP-enriched stock. Figure 2C shows a plot of DIP stock (in μL) versus % inhibition of TKHA expression from STD PIV5. It is clear that this particular stock had a functional unit of PIV5 DIP, which corresponded to 21.3 μL of DIP-enriched stock needed to reduce STD PIV5-TKHA-infected cells by 50% in a cell population of 60,000 cells.

This value, defined as DI units, provided a standardized basis for adjusting DIP delivery across experiments. The interference capacity of DIPs was further confirmed by Western blotting for TKHA expression in samples from A549 cells infected with STD PIV5-TKHA and increasing levels of DIP-enriched PIV5 stock (Figure 2D). The differences observed in total protein levels for P and TKHA could be due to variations in their translation efficiency, stability, or degradation rates.

**Figure 3:**
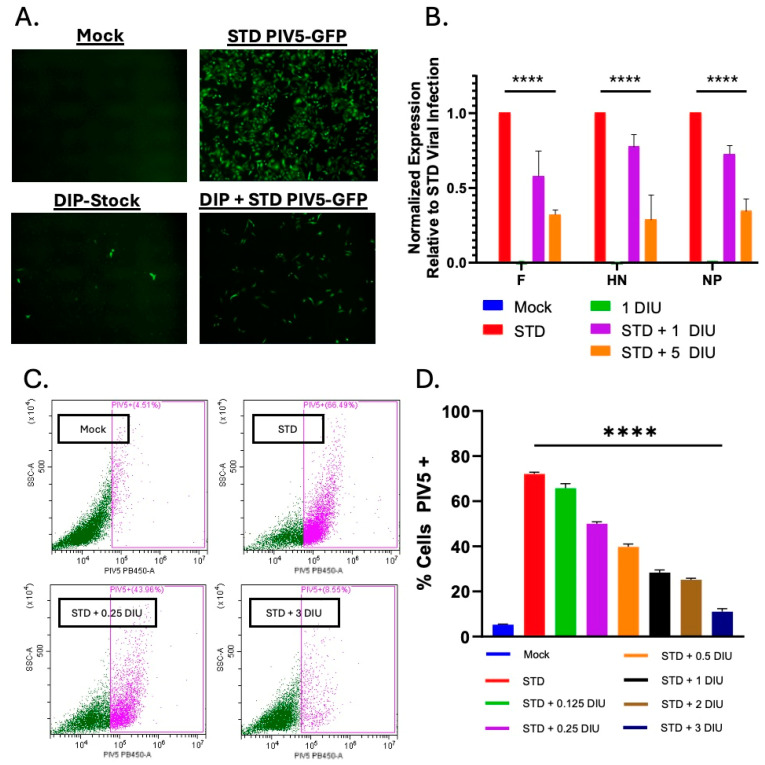
PIV5 DIPs suppress STD virus gene expression and reduce expression of PIV5 surface glycoproteins during coinfection. (**A**) A549 cells were mock-infected, or infected with STD PIV5-GFP at an MOI of 10, DIP-enriched stock (10 DI units) alone, or co-infected with STD PIV5-GFP and DIP-enriched stock (10 DI units). Cells were examined for GFP expression at 18 hpi with fluorescence (FL) microscopy (**A**). (**B**) A549 cells were mock-infected, or infected with STD PIV5, 1 DIU alone, or STD PIV5 and either 1 or 5 DIUs. At 18 hpi, RNA was isolated and analyzed by quantitative PCR analysis for levels of PIV5 F, HN and NP mRNA. Data represent the relative expression of each viral gene normalized to a GAPDH housekeeping gene. (**C**) Representative scatter plot images comparing PIV5 surface glycoprotein levels for mock-infected vs. STD PIV5 infected vs. STD PIV5 and DIU (0.25, 3) infected cells by flow cytometry (**D**) A549 cells were mock-infected, or infected with STD PIV5 alone, or STD PIV5 with increasing doses of DIUs (0.125, 0.25, 0.5, 1, 2, 3). At 18 hpi, cells were analyzed for surface levels of PIV5 proteins by flow cytometry. Data represent the percentage of cells in a population positive for the PIV5 glycoproteins. Values in all panels are the means of three replicates, with error bars representing standard deviation. **** indicates a *p* value < 0.0001.

To further visualize the interference capability of DIPs, we used an STD PIV5 that expresses GFP for live cell imaging. A549 cells were mock-infected, or infected with STD PIV5-GFP alone, DIP-enriched stock alone, or co-infected with STD PIV5-GFP and DIPs (10 DI units). As shown in Figure 3A, cells infected with STD PIV5-GFP-infected cells exhibited high-level GFP expression. By contrast, infection with DIP-enriched PIV5 showed very few cells expressing bright GFP, and co-infections of STD PIV5-GFP with DIP-enriched stock (10 DI units) resulted in a significant reduction in GFP-expressing cells, compared to STD virus infection alone.

Viral surface glycoproteins can be a trigger for C′-mediated killing of infected cells [52,53,54,55,56]. To determine the effect of DIP infection on STD PIV5 glycoprotein expression, A549 cells were mock-infected, infected with STD PIV5-GFP, or coinfected with STD PIV5-GFP along with 1 DI unit or 5 DI units of DIPs. Levels of viral mRNA were analyzed by qPCR. As shown in Figure 3B, 5 DI units were sufficient to reduce the expression of STD PIV5 nucleoprotein (NP), Hemagglutinin-Neuraminidase (HN), and Fusion (F) mRNAs to ~25% of control samples lacking DVGs. In addition to mRNA changes, the effect of DIPs on PIV5 surface glycoprotein levels were examined using an anti-PIV5 antibody and flow cytometry. As shown in representative Figure 3C, infection with STD PIV5 resulted in strong cell surface staining for PIV5 glycoproteins at 67% of cells. Importantly, the cell surface expression of PIV5 glycoproteins showed a dose-dependent decrease with increasing co-infection with DI units (Figure 3D), and 1 DI unit was sufficient to reduce surface staining to ~50% of that seen with STD virus alone. 

### 3.3. Infection with DIP-Enriched Stocks Reduces C′-Mediated Killing of Cells Infected with STD PIV5

Given that C′ is highly effective in lysing cells infected with STD PIV5 virus [39,40], we tested the hypothesis that DIP co-infection with STD PIV5 would alter C′-mediated cell lysis. Our previously described real-time cell monitoring assay was utilized to define the kinetics of C′-mediated killing of PIV5-infected lung cells with or without co-infection by DIP [39,40]. Using A549 cells that stably express a nuclear red fluorescent protein (A549-NLR), the IncuCyte instrument can record red fluorescence nuclei in real-time and at defined continuous intervals. A549-NLR cells were mock-infected, infected with STD PIV5-GFP at an MOI of 10, infected with 10 DI units alone, or co-infected with STD PIV5 and 10 DI units. At 18 hpi, cells were treated with 10% NHS as a source of active C′ factors. Every 2 h, the number of red-labeled cells (Red Object Counts, ROC) was determined per well and normalized to the ROC at time 0 (ROCt0). Data were expressed as a percentage ROC of time zero when NHS was added (ROC/ROCt0).

**Figure 4:**
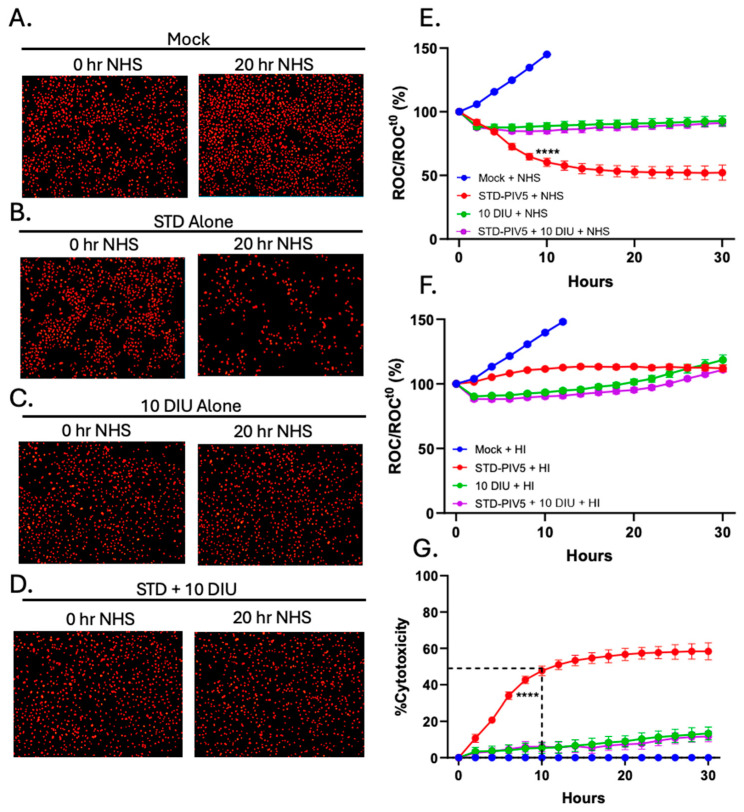
DIP-enriched PIV5 infections blocked C′-mediated killing of A549 lung epithelial cells. (**A**–**G**) A549-NLR cells were mock-infected, STD PIV5 infected at an MOI of 10, infected with 10 DI units, or co-infected with PIV5 STD at an MOI of 10 and 10 DI units. At 18 hpi, each condition was treated with NHS at 10% as a source of C′ factors. (**A**–**D**) Representative red fluorescent images of A549-NLR cells incubated with NHS were recorded using the IncuCyte instrument at 0 and 20 h post NHS addition. (**E**) Red object count (ROC) per well was quantified using the IncuCyte instrument and normalized to the ROC at time zero (ROC/ROCt0) when NHS was added, expressed as a percentage of time zero. (**G**) Percentage cytotoxicity was calculated from the data in panel (**E**,**F**) as described in Materials and Methods. Values are the mean of three replicates with error bars representing standard deviation; **** indicates when a *p*-value of <0.001 first appears in the time course comparing STD PIV5 infected to 10 DI unit or STD PIV5 + 10 DI unit infected cells incubated with NHS. This statistical significance was maintained throughout the remainder of the time course.

Representative images taken 0 and 20 h after NHS addition are shown in Figure 4A–D. For mock-infected cells, the number of red-labeled cells increased over time, consistent with cells continuing to grow and with no lysis by NHS addition (panel A). By contrast, cells infected with STD PIV5 showed a loss of the majority of red-labeled cells by 20 h after adding NHS (panel B), as shown in our previous work [39,40]. Infection with DIP alone did not decrease the number of red-labeled cells (panel C), and most importantly, cells co-infected with STD PIV5 and DIPs showed no loss of cells with added NHS (panel D). The real-time kinetics of C′-mediated cell killing for these samples is shown in Figure 4E, expressed as a percentage of the initial time zero when NHS addition occurred (ROC/ROCt0(%)). Cells infected with STD PIV5 showed significant loss of ROC with added NHS (red line). Importantly, co-infection with 10 DI units was sufficient to completely block C′-mediated killing of STD PIV5-infected cells (purple line). HI NHS did not alter cell killing in all conditions (Figure 4F).

This quenching of C′-mediated killing of cells infected with STD PIV5 by DIPs is clearly seen when these same data are plotted in Figure 4G as a percent cytotoxicity—C′-mediated killing for each condition was normalized to values with HI NHS (panel F) to account for differences in cell growth during culturing. Here, C′-mediated killing of STD PIV5-infected cells reached 50% of time zero value by 10 h after NHS addition (Figure 4G, red line). By contrast, the addition of DIP to STD PIV5 infections reduced cytotoxicity to 10% or less (purple line).

**Figure 5:**
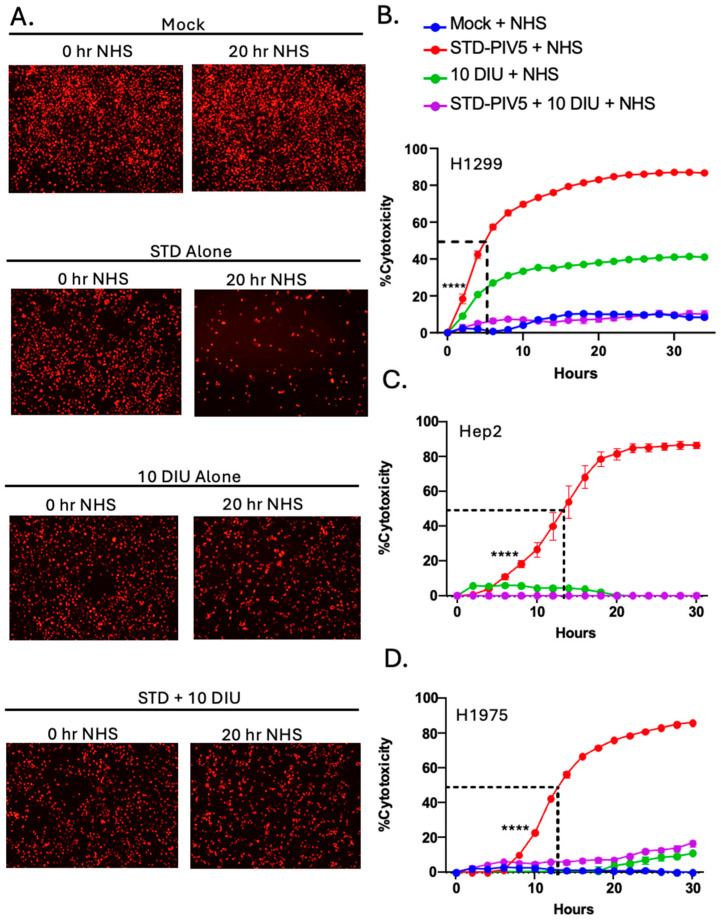
C′-mediated killing of PIV5-infected H1299, Hep2, and H1975 cells is reduced by co-infection with DIP-enriched stocks. (**A**–**D**) H1299-NLR, Hep2-NLR, and H1975-NLR cells were mock-infected, or infected with STD PIV5 at an MOI of 10, 10 DI unit alone, or co-infected with STD PIV5 and 10 DI unit. At 18 hpi, samples were treated with 10% NHS as a source of C′ factors. (**A**) Representative red fluorescent images of H1299-NLR cells incubated with NHS were recorded using the IncuCyte instrument at 0 and 20 h after NHS addition. (**B**–**D**) For each cell type, ROC recorded for samples treated with NHS was normalized to the ROC of cells in the absence of NHS as described for the data in Figure 4 above. Values are the mean of three replicates with error bars representing standard deviation; **** indicates when a *p*-value of <0.001 first appears in the time course comparing STD PIV5-infected and NHS-treated samples versus DIP alone and NHS-treated, or co-infected STD PIV5 + DIP and NHS-treated samples.

The effect of DIP co-infection on C′-mediated killing was analyzed in three additional human lung cell lines—H1299, Hep2, and H1975. H1299-NLR, Hep2-NLR, or H1975-NLR cells that were stably transduced to express NLR for real-time tracking by the IncuCyte were mock-infected, or infected with STD PIV5-GFP at an MOI of 10, with 10 DI units only, or co-infected with STD PIV5 and 10 DI units. At 18 hpi, cells were treated with 10% NHS as a source of C′. Figure 5A shows representative images taken 0 and 20 h post NHS addition for H1299-NLR cells, illustrating a loss of ROC for NHS-treated STD PIV5 infections and a quenching of C′-mediated lysis with DIP co-infection. As shown in Figure 5B,D, C′-mediate cytotoxicity was faster for STD PIV5-infected H1299 cells infected (red lines) with a 50% cytotoxicity at 5 h post NHS treatment compared to Hep2 and H1975 cells where 50% cytotoxicity seen at ~12 h post treatment. Most importantly, the addition of DIP during infection with STD PIV5 reduced C′-mediated killing of all three PIV5 infected human lung cell lines.

### 3.4. DIP Inhibition of C′-Mediated Killing of PIV5 Cells Is Dependent on DIP Replication but Independent of IFN-I Induction

**Figure 6:**
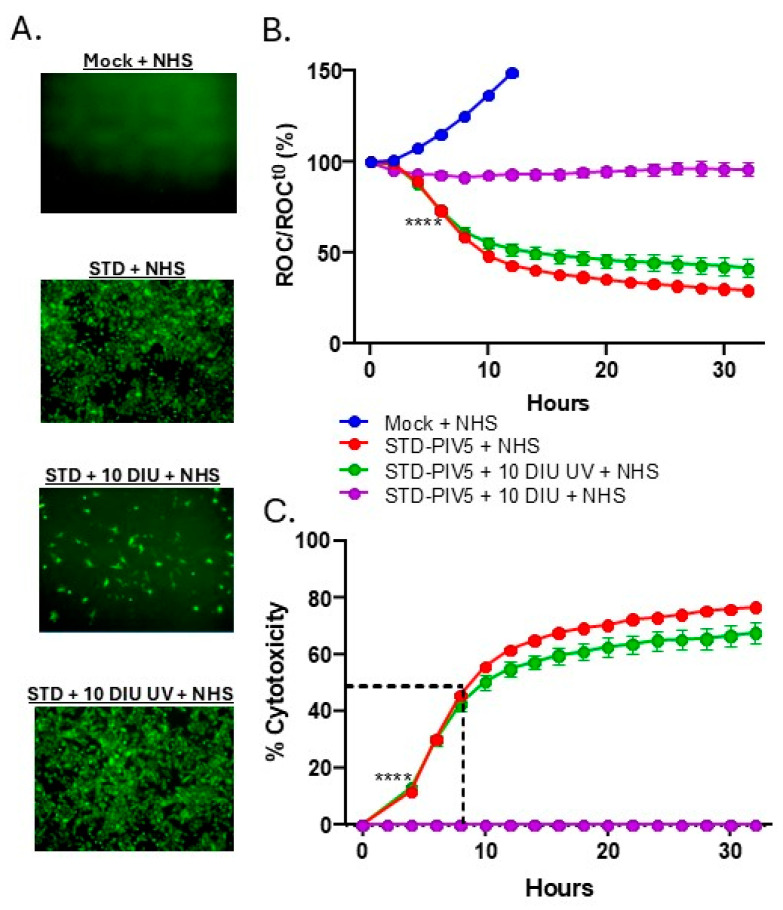
UV treatment of DIP-enriched PIV5 reduces interference with C′-mediated killing of STD PIV5-infected cells. (**A**–**C**) DIP-enriched PIV5 stock was UV treated for 15 min prior to infections. A549-NLR cells were mock-infected, infected with STD PIV5 at an MOI of 10, or co-infected with STD PIV5 and 10 DI unit, either UV-treated or UV-untreated. (**A**) Representative images showing GFP expression of cells at 18 hpi with FL microscopy. (**B**) ROC for A549-NLR cells per well was quantified over the indicated times using the IncuCyte instrument and normalized to the ROC at time zero (ROC/ROCt0) when NHS was added. (**C**) Percentage cytotoxicity was calculated from the data in panel B as described in Materials and Methods. Values are expressed as a percentage of time zero, and are the mean of three replicates, with error bars representing standard deviation; **** indicates when a *p* value < 0.0001 first appeared on the time course, comparing STD PIV5 infection or STD PIV5 + UV-treated 10 DI unit infected cells incubated with NHS to STD PIV5 + 10 DI unit UV-untreated infected cells incubated with NHS.

To determine if the ability of DIPs to inhibit C′-mediated lysis of PIV5 infected cells depended on DVG replication, a DIP-enriched stock was UV-treated for 15 min under conditions which completely inactivate PIV5 replication [57]. A549 cells were then mock-infected, infected with STD PIV5-GFP at an MOI of 10, or co-infected with STD PIV5-GFP and 10 DI units UV-treated or untreated. At 18 hpi, NHS was added as a source of C′ to a final concentration of 10%. As shown in the fluorescence micrographs in Figure 6A, cells co-infected with DIPs and STD PIV5 showed strong inhibition of GFP expression, as shown above. In sharp contrast, UV-treated DIPs did not alter GFP expression during co-infection with STD PIV5.

The effect of UV-treated DIP co-infection on C′-mediated killing of PIV5 infected cells was examined using the real-time IncuCyte instrument. As shown in Figure 6B, mock-infected cells showed continued growth after NHS addition (blue line), while cells infected with STD PIV5-GFP alone showed a significant 50% reduction in cell viability compared to baseline following NHS addition (red line), reflecting the effectiveness of C′-mediated killing in the absence of DIPs. In co-infections with STD PIV5-GFP and UV-untreated DIPs, C′-mediated lysis of cells was nearly equivalent to lysis of cells with STD PIV5 alone (green line). Figure 6C shows the same data plotted as a percentage of cytotoxicity, emphasizing that the kinetics and extent of C′-mediated lysis of cells infected with STD PIV5 was not significantly altered by co-infection with UV-treated DIPs.

**Figure 7:**
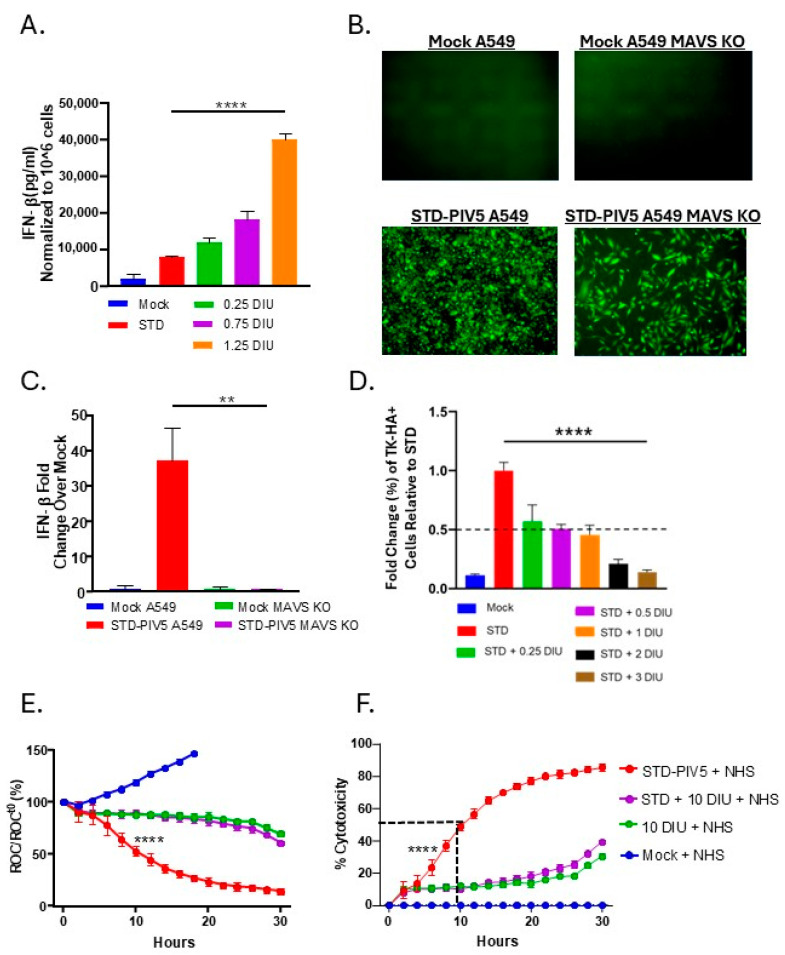
DIP reduction in C′-mediated killing of PIV5-infected cells is independent of IFN induction. (**A**) A549 cells were mock-infected, or infected with STD PIV5 at an MOI of 10, or infected with DIP-enriched stock with increasing DI units (0.25, 0.75, 1.25). At 18 hpi, media were analyzed for IFN- β levels by ELISA. (**B**,**C**) A549 or A549-MAVS-KO-NLR cells were mock-infected, or infected with STD PIV5 an MOI of 10. At 18 hpi, GFP levels were monitored by fluorescent microscopy and media analyzed for levels of IFN-β by ELISA. (**D**) A549-MAVS-KO cells were infected with STD PIV5-TKHA alone at an MOI of 10, or coinfected with the indicated units of DIP-enriched stock along with STD PIV5-TKHA. Levels of intracellular TKHA were determined by flow cytometry at 18 hpi. (**E**,**F**) A549-MAVS-KO-NLR cells were mock-infected, or infected with STD PIV5 at an MOI of 10, 10 DI unit alone, or co-infected with STD PIV5 and 10 DI unit. ROC for A549-MAVS-KO-NLR cells per well was quantified using the IncuCyte instrument and normalized to the ROC at time zero (ROC/ROCt0) when NHS was added. (**F**) Percent cytotoxicity was calculated from the data in panel E as described in materials and methods. Values are expressed as a percentage of time zero and are the mean of three replicates, with error bars representing standard deviation; **** indicates when a *p* value < 0.0001 first appeared on the time course, comparing STD PIV5-infected cells to 10 DI unit or STD PIV5 + 10 DI unit-infected cells incubated with NHS.

DVGs are known to be strong inducers of IFN-I [27,58,59,60]. Therefore, we tested the hypothesis that DVG-induced IFN-I was responsible for the inhibition of C′-mediated killing of STD PIV5-infected cells. In theory, this could occur through autocrine signaling by IFN-I to indirectly suppress viral gene expression necessary for C′ activation or by crosstalk between IFN-I and C′ pathways. To confirm that our DIP-enriched stock induced significant levels of IFN-β compared to a STD PIV5 infection, media were harvested at 18 hpi from A549 cells infected with STD PIV5-GFP alone or increasing DI units of DIP and analyzed by ELISA for levels of IFN-β. As shown in Figure 7A, STD PIV5-GFP infection induced relatively low levels of IFN-β, and this was increased in a dose-dependent manner by DIP infections. We utilized A549-MAVS-KO cells, which lack a key signaling factor involved in IFN-β induction, as our cellular model to evaluate the impact of DVG-induced IFN-β on C′-mediated killing. A549-MAVS-KO cells were equally as susceptible to infection by STD PIV5 as parental A549 cells (Figure 7B), and showed reduced background levels of IFN-β induction after STD PIV5 infection (Figure 7C). To determine whether DIPs maintain their interference capacity in A549-MAVS-KO cells, cells were mock-infected, or infected with STD PIV5-TKHA, at an MOI of 10 alone or as co-infection with increasing DI units of DIPs. As seen in Figure 7D, A549-MAVS-KO cells showed a dose-dependent decrease in TKHA expression from STD PIV5 expression, with one DI unit inhibiting 50% of TK expression. These data indicate that DIPs can still successfully interfere with STD PIV5 gene expression in the absence of IFN-I production.

The ability of DIPs to reduce C′-mediated killing of PIV5-infected A549-MAVS-KO-NLR cells was analyzed by real-time killing assays with the IncuCyte instrument. Cells were mock-infected or infected with STD PIV5-GFP alone, 10 DI units alone, or co-infected with STD PIV5-GFP and 10 DI units were treated with 10% NHS at 18 hpi. Cell death was monitored over time. As shown in Figure 7E, cells infected with STD PIV5 were killed with a 50% point at ~10 hp treatment, similar to the data in Figure 4E for the parental A549 cells. Importantly, DIP-enriched infections showed reduced C′-mediated killing of cells compared to STD PIV5 infected cells (green and purple lines). Figure 7F shows the same data plotted as a percentage of cytotoxicity, highlighting that the reduction in C′-mediated killing by DIP co-infection occurs independent of IFN-I production, with kinetics of killing that match parental A549 infected cells (see Figure 4G above).

### 3.5. Linear Relationship Between DIP-Mediated Changes in Cell Surface Glycoprotein Expression and C′-Mediated Killing

**Figure 8:**
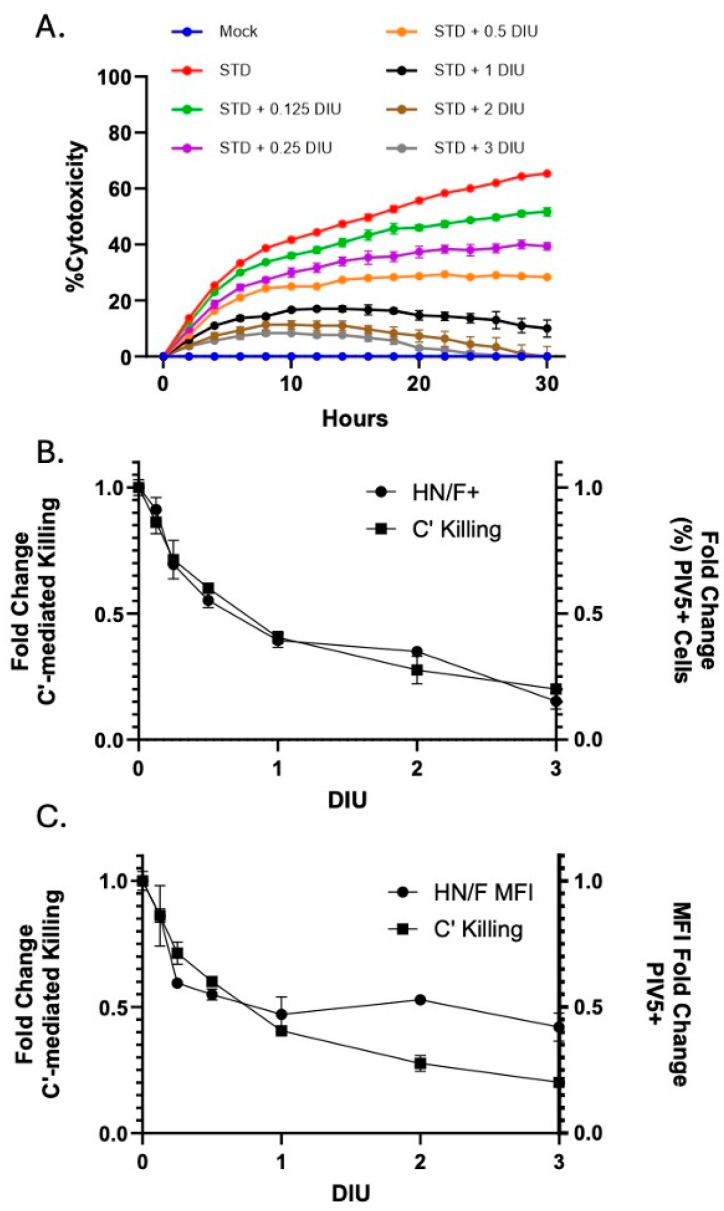
Relationship between the effect of DIP co-infection on PIV5 glycoprotein expression and C′-mediated killing. A549-NLR cells were co-infected with STD PIV5 and increasing doses of DI units (0.125, 0.25, 0.5, 1, 2, 3 units). At 18 hpi, cells were analyzed for PIV5 glycoprotein surface expression by flow cytometry. In parallel, cells at 18 hpi were treated with NHS and monitored by IncuCyte for C′-mediated killing post NHS addition. (**A**) Percentage cytotoxicity was calculated for increasing DI unit dosage as described in Materials and Methods. (**B**,**C**) This approach enabled the assessment of C′-mediated killing in cell populations exhibiting dose-dependent differences in glycoprotein expression at the time of NHS treatment. Across the DIU dosage titration (*x*-axis), the dual-axis plots illustrate the relationship between C′-mediated killing at 10 h post NHS addition (left *y*-axis) and either a fold change in the percentage of cells in the population that are positive for PIV5 glycoprotein surface expression relative to STD PIV5 (panel (**B**)) or MFI PIV5+ cells relative to STD PIV5 (panel (**C**)) (right *y*-axis).

To assess the potency of DIP-enriched stocks to inhibit C′-mediated lysis of PIV5-infected cells, we performed a dose titration where A549 cells were infected with STD PIV5-GFP along with increasing DI units. At 18 hpi, cells were treated with NHS to a final concentration of 10% and killing was monitored by the real-time IncuCyte instrument. Figure 8A depicts the kinetics of C′-mediated cytotoxicity versus increasing DIP dose. At lower DI unit doses (0.125, 0.25), only a slight reduction in C′-mediated killing is observed (compare red, green, and purple lines). However, as the DI unit concentration increases (0.5, 1, 2, 3 units), the inhibition of cytotoxicity becomes more pronounced (compare orange, black, and brown lines).

Figure 8B shows the relationship between DI-induced changes in viral surface glycoprotein expression and C′-mediated killing of infected cells. Titration of DI units in co-infection with STD PIV5 infection showed a strong linear relationship between decreasing surface viral glycoprotein expression and the inhibition of C′-mediated lysis. As shown in Figure 8C, very similar relationships were seen between changes in the cell surface mean fluorescent intensity (MFI) of viral glycoproteins and the inhibition of C′-mediated lysis. Together, these data support the contention that a major mechanism for the inhibition of C′-mediated cell killing is through the depletion of viral surface glycoprotein expression.

## 4. Discussion

DVGs have emerged as integral components of many naturally occurring RNA virus populations [61], with growing evidence of their ability to modulate both virus gene expression as well as host immunity [34,62,63,64,65,66,67,68,69]. Using the prototypic non-segmented negative-strand RNA virus PIV5, we have generated DIP-enriched stocks to test the hypothesis that DVGs can alter C′-mediated lysis of PIV5-infected cells. Here, we demonstrate that during co-infection of human lung cells in culture with STD PIV5, DIPs show a potent inhibitory effect on C′-mediated cell lysis which is both dose- and replication-dependent, but independent of their ability to induce IFN-I. To our knowledge, this is the first report of contributions of negative-strand RNA virus DVGs to altered C′ activities.

DIPs are typically characterized as strong inducers of IFN-I [46], as shown here for the PIV5 DIPs analyzed in lung epithelial cells. There are reported examples of crosstalk between C′ and IFN-I pathways, which can result in changes in expression of different IFN and C′ factors or their activities [70,71]. In our model system based on lung cancer cell lines, we found that the ability of DIP co-infection to reduce the C′-mediated lysis of PIV5-infected cells was not changed when the host cell had a genetic knockout of the key IFN-I component MAVS. While a functional crosstalk between these two innate pathways may be evident in more complex cell cultures or in vivo, our results do not support IFN-I induction as a critical feature of the mechanism of DIP inhibition of C′.

Negative-strand RNA viruses frequently generate copyback genomes which contain two copies of the strong viral antigenomic promoter [72,73], and it is thought that competition for trans-acting viral factors (e.g., polymerase) is the main mechanism for the DIP interference in STD virus growth. Importantly, this interference is also seen at the level of DVG-mediated decreases in STD viral protein levels [74]. This is consistent with our findings that UV inactivation of DIPs removed their ability to inhibit C′-mediated lysis of STD PIV5-infected cells and supports the proposal that the ability to interfere with STD virus gene expression is key to altering susceptibility to C′-mediated lysis.

Prior studies have shown that the HN and F glycoproteins of negative-strand RNA viruses, or their associated activities, are recognized by the C′ system [52,53,55]. Similarly, single amino acid substitutions that alter F protein activity can dramatically alter C′ pathways and activity [56]. We found that the inhibition of C′-mediated killing by DIPs showed a direct correlation with a reduction in levels of PIV5 surface glycoproteins, both within the overall cell population (e.g., percentage of positive staining) and on individual cells (MFI). A key to understanding this relationship was in our careful defining of a DI unit for PIV5 DIPs, and its use in normalizing what effectively becomes a “multiplicity of infection” with DIPs. This seemingly straightforward relationship between DIP effect on C′-mediated lysis and the cell surface expression of viral glycoproteins may not reflect the simple inhibition of HN and F expression at the level of mRNA production. For example, DVGs could also affect HN and F glycoproteins differently, possibly disrupting their glycosylation, intracellular trafficking, and turn-over rate, as shown previously for the effect of Sendai virus DVGs [75]. Further exploration is needed to determine whether DVGs differentially affect HN or F and to explore their individual contribution to the sensitivity of lysis of virus-infected cells.

One limitation of our study is that, while different DIP-enriched stocks were titrated to the same DI unit, the exact genomic composition of both STD PIV5 and DVGs was not fully characterized. However, the consistent results in C′ killing and surface glycoprotein changes across multiple stocks suggest these variations did not significantly impact our findings. Future research should focus on a more detailed analysis of the STD PIV5 and DVGs within individual stocks to better understand their potential influence.

In our H1299 cell line, infection with DIPs alone caused C′-mediated killing, although at a lower level than STD PIV5 infection. This raises an important question: why do some cells undergo lysis while others do not, particularly in DIP-enriched infections? One possibility is that the susceptibility of individual cells to C′-mediated lysis may be influenced by differences in DVG content. Cells that accumulate higher DVG levels may exhibit distinct characteristics compared to those with lower DVG content, potentially affecting their ability to be lysed by C′. Additionally, it is possible that certain cells within a population are inherently different from one another, leading to stress-induced pathways that allow C′-killing. Exploring these individual cell differences could provide valuable insights into why certain cells are resistant to lysis, while others are effectively targeted by C′. While our results clearly show that DVGs reduce C′-mediated killing in the short term, it is also important to consider the long-term fate of these DVG-containing cells. Although DVGs seem to offer immediate protection by evading immune clearance, this effect may not persist indefinitely. It is possible that, despite the reduction in C′ activation, DVG-infected cells may still encounter additional host cell checkpoints or stress responses that ultimately lead to cell death over time. Future studies should address these aspects, potentially focusing on the extended survival of DVG-infected cells.

DVGs are frequently observed in clinical isolates from patients with chronic or persistent viral infections, raising important questions about DVGs in prolonging the clearance of a viral infection [10,14,17,18,19,20,21]. One study found that cells enriched in DVGs prevented TNF-alpha-mediated cell death by enacting a pro-survival mechanism dependent on MAVS signaling [36,61]. Our work demonstrates how DVG-induced blocking of C′ responses may provide a mechanism by which viruses evade host immunity, enabling prolonged infection. This could lead to significant interference by DVGs, potentially reducing viral protein levels below the threshold required to activate C′ and trigger cell death.

Our findings also have implications for live-attenuated vaccines, which in some cases have been shown to harbor DIPs [23,24,25,26]. If our findings on the DIP suppression of C′-mediated immune responses hold true for clinical situations, their presence in vaccines could potentially compromise vaccine efficacy, especially for those immune responses that are heavily influenced by the C′ system [76,77]. Likewise, after the delivery of attenuated viruses, the presence of DVGs might delay the eventual clearance of the live viral vaccine by C′-mediated mechanisms. With a standardized DI unit, we can now precisely quantify the amount of DIPs needed to interfere with viral replication. By determining the threshold at which DIPs effectively reduce viral load without undermining vaccine efficacy, optimized vaccine formulations could be produced. These observations underscore the importance of monitoring DIP content during vaccine production and assessing their potential impact on vaccine-induced immune responses or residual viral load following treatment. 

## Figures and Tables

**Table 1:** Nucleotide primers used for q-PCR.

Name	F′ Primer	R′ Primer
GAPDH	5′-TTAAAAGCAGCCCTGGTGAC-3′	5′-CTCTGCTCCTGTTCGAC-3′
F	5′-ACGTGTTATGGTGACTGGCA-3′	5′-GAACAGCACGAATCGAGTGA-3′
HN	5′-TGACCAACCCTTCGTCTACC-3′	5′-CTTGACCGCTTGATCCAAAT-3′
NP	5′-TGACCAGTCACCAGAAGCTG-3′	5′-CGGAATCAACGAAAGGTGTT-3′

## Data Availability

The original contributions presented in this study are included in the article. Further inquiries can be directed to the corresponding author.
